# Atypical CT Findings in Plexiform Ameloblastoma

**DOI:** 10.1155/2014/623093

**Published:** 2014-11-24

**Authors:** Karandeep Singh Arora, Nagesh Binjoo, Richa Modgil, Lalit Singh Negi, Prabhpreet Kaur

**Affiliations:** ^1^Department of Oral Medicine & Radiology, Rajasthan Dental College & Hospital, N.H. 8, Ajmer Road, Bagru Khurd, Jaipur, Rajasthan 302026, India; ^2^Department of Oral Medicine & Radiology, DR. B.R. Ambedkar Institute of Dental Sciences & Hospital, Patna, Bihar 801503, India; ^3^Department of Oral & Maxillofacial Pathology, Darshan Dental College & Hospital, Udaipur, Rajasthan 313001, India

## Abstract

Ameloblastoma is an uncommon epithelial odontogenic neoplasm that is nonmineralized, locally aggressive, and, in most cases, benign. Most ameloblastomas develop in the molar-ramus region of the mandible with 70% of them arising in the molar-ramus area. Radiologically they are unilocular or multilocular radiolucency with a honeycomb or soap bubble appearance. The radiographic appearance of ameloblastoma can vary according to the type of tumor. CT is usually helpful in determining the contours of the lesion, its contents, and its extension into soft and hard tissues. Through this case we would bring to light some of the unusual CT findings which include the destruction of the surrounding structures by the lesion which appeared to be normal routine lesion when viewed clinically.

## 1. Introduction

Ameloblastoma is an uncommon epithelial odontogenic neoplasm that is nonmineralized, locally aggressive, and, in most cases, benign. Ameloblastoma accounts for approximately 10% of all tumors that originate in the maxilla and mandible [1]. The suggested aetiology of ameloblastoma is that it either arises from the dental lamina or more probably, it arises from basal cells of the oral epithelium or from cells that have undergone differentiation to mimic the ameloblast [2]. Most ameloblastomas develop in the molar-ramus region of the mandible with 70% of them arising in the molar-ramus area and they are occasionally associated with unerupted third molar teeth. The chief histopathological variants of ameloblastoma are the follicular and plexiform types, followed by the acanthomatous and granular cell types. Uncommon variants include desmoplastic, basal cell, clear cell ameloblastoma, keratoameloblastoma, and papilliferous ameloblastoma. It is well known that ameloblastoma can be radiologically unilocular or multilocular radiolucency with a honeycomb or soap bubble appearance [3]. The purpose of reporting this case is to bring to light the CT findings that seem to be unusual. This case is being sent forwards after the prior approval of the Institutional Review Board.

## 2. Case Report

A 27-year-old male patient reported to the Department of Oral Medicine & Radiology, with a complaint of asymmetric swelling on the lower left jaw. Patient stated that swelling was gradual on onset and progressed in size in a 2-year course. There is history of pus discharge from the lower left back teeth region since 2 years. He got his lower left back tooth extracted 10–12 years back and since then there is an unhealed extraction socket for which patient had consulted several dental practitioners by whom he was treated unsuccessfully without investigations and proper diagnosis. Patient was physically healthy and mentally alert.

Extra orally there was ill defined solitary spherical swelling involving the left angle of mandible measuring 7 × 5 cm which was noncompressible, nonreducible, nonmobile, nonfluctuant, and fixed to the underlying structure with a notch in the body of mandible 3 cm anterior from the angle of mandible which was soft and fluctuant and there was deviation of jaw on the right side. Intraorally buccal plate expansion was felt from region of tooth 35 to the angle of mandible and the overlying mucosa was normal with egg shell crackling near region of tooth 37. Lingual cortical plate expansion in teeth regions of 37 and 38 and a yellow color discharge from socket of tooth 38 and root stump of tooth 36 were present. Pulp vitality test of tooth 37 revealed a nonvital tooth. Clinical provisional diagnosis established was ameloblastoma of left mandible with a differential diagnosis of residual cyst, dentigerous cyst, keratocystic odontogenic tumour, central giant cell granuloma, and radicular cyst.

**Figure 1:**
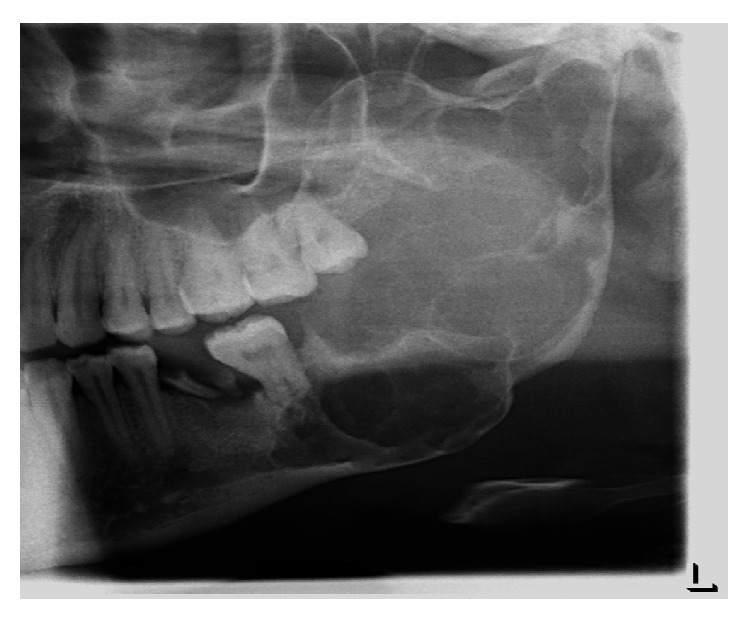
OPG of the involved site showing areas involved and bone destruction.

**Figure 2:**
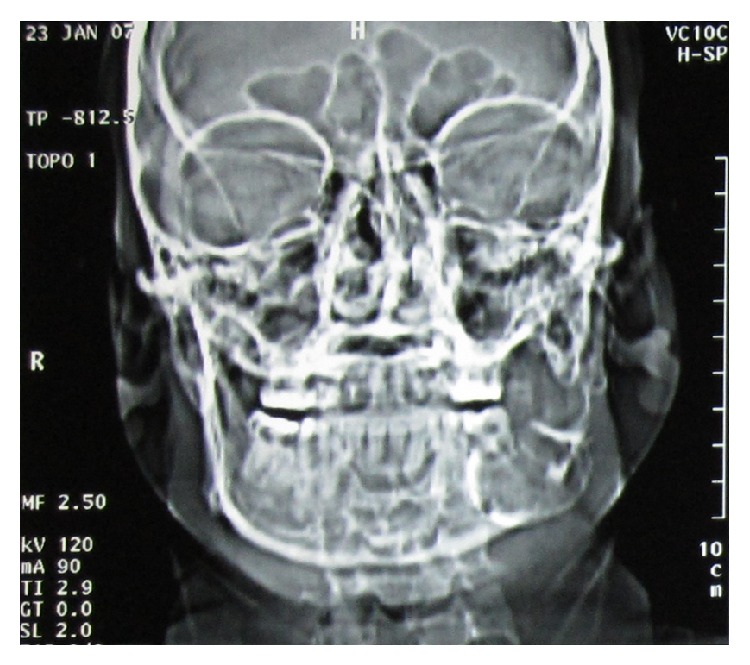
Coronal section of CT with visible buccal bone expansion.

IOPAR (intraoral periapical radiograph) revealed a radiolucency extending out from the boundaries of IOPA film. OPG (orthopantomogram) (Figure 1) revealed a multilocular radiolucency having scalloped and hyperostotic borders involving the left side of mandible extending from posterior body involving the angle, ramus, coronoid process, and the condyle of the ipsilateral side with downward expansion of inferior border of mandible. A relatively dark radiolucency is seen below tooth 37 measuring 3 × 2.5 cm suggestive of window formation. Resorption of mesial and distal root of tooth 37 is also seen. CT scan image (Figure 2) revealed involvement of the left mandibular coronoid process, condyle, ramus, and body. Unusual finding of CT imaging at the level of middle 1/3rd of ramus revealed deflection of left lateral pterygoid plate and at the level of maxillary alveolar crest it revealed deflection of left maxillary posterior alveolar arch towards the midline. These findings were evident because of medial expansion of ramus. The radiographic diagnosis given was ameloblastoma.

Soft tissue incisional biopsy from unhealed socket of tooth 38 was suggestive of ameloblastoma. Selective resection of mandible was done in Department of Oral & Maxillofacial Surgery under general anaesthesia and excised sample was sent for histopathological examination where diagnosis was confirmed to be ameloblastoma of plexiform type.

## 3. Discussion

Although the term ameloblastoma was coined by Churchill in 1933, the first detailed description of this lesion was by Falkson in 1879 [4].

The ameloblastoma usually occurs in persons between the age of 20 and 50 years with the average age being 39 years. About 80% occur in the mandible and the remainder in the maxilla [2].

Radiologically, the lesions are expansile, with thinning of the cortex in the buccal-lingual plane. The lesions are classically multilocular and cystic with a “soap bubble” or “honeycomb” appearance. On occasion, conventional radiographs reveal unilocular ameloblastomas, resembling dentigerous cysts, or odontogenic keratocysts. The radiographic appearance of ameloblastoma can vary according to the type of tumor. CT is usually helpful in determining the contours of the lesion, its contents, and its extension into soft tissues [5].

Ameloblastomas are treated by curettage, enucleation plus curettage, or radical surgery. [6, 7] Comparing long-term results for 78 ameloblastomas, Nakamura and others reported that the rate of recurrence is 7.1% after radical surgery and 33.3% after conservative treatment [7]. In their series of 26 ameloblastomas, Sampson and Pogrel showed that nearly 31% of tumours recurred after conservative surgery. In their study, they treated 3 patients with enucleation and bone curettage and 1 patient with hemimandibular resection. In 3-year follow-up, there has been no recurrence of the tumours [8].

**Figure 3:**
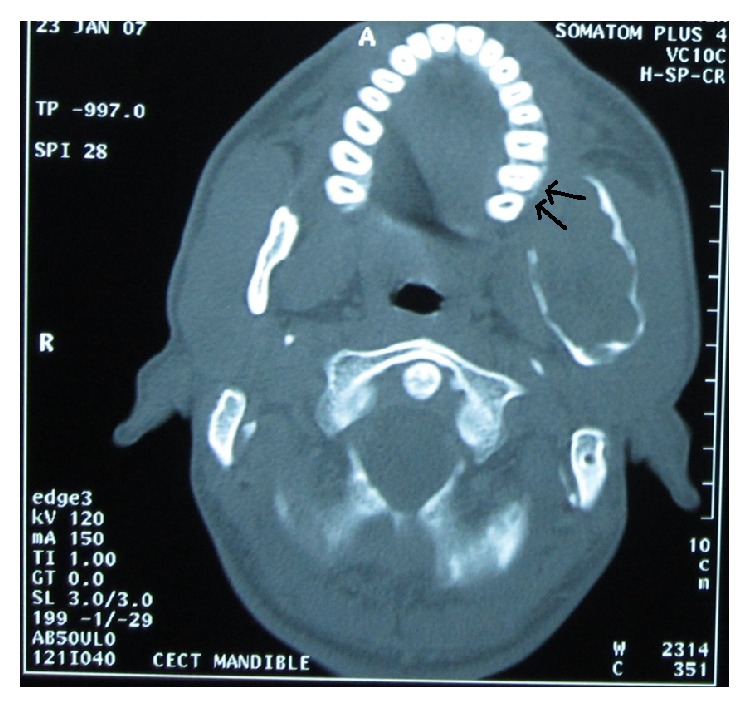
Transverse section of CT showing deflection of the left maxillary arch medially near the tuberosity.

**Figure 4:**
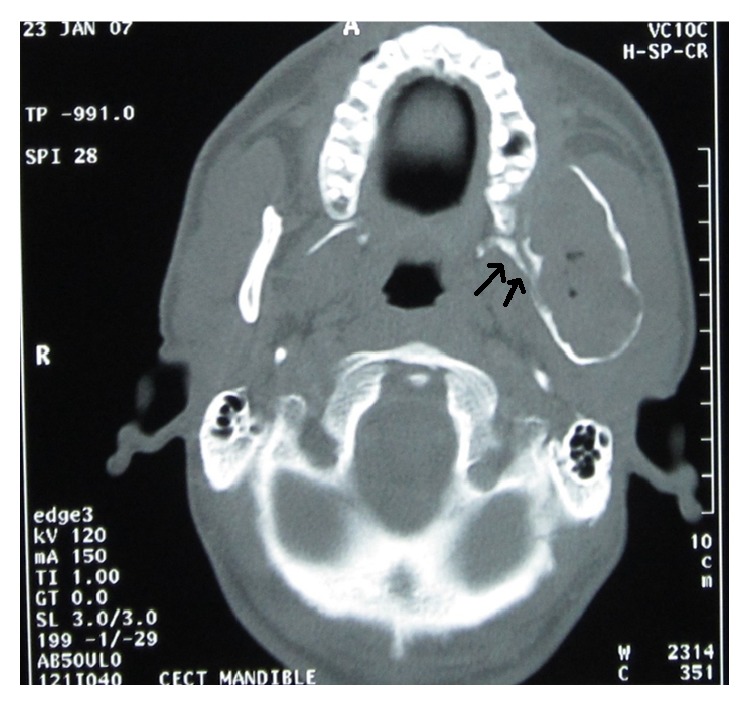
Transverse section of CT showing deflection of the left lateral pterygoid plate medially also with the left maxillary alveolar crest.

The tumor found in our patient was an ameloblastoma of the plexiform type. The term “plexiform” refers to the appearance of anatomizing islands of odontogenic epithelium in contrast to a follicular pattern. The tumor was found to be extending from mandibular body up to ramus, coronoid process, and condyle. It was associated with unhealed extraction socket since 10 years. The unusual CT findings significantly were as follows: firstly, there was deflection of left maxillary posterior arch towards the midline because of medial expansion of ramus (Figure 3). Secondly, there is deflection of lateral pterygoid plate towards midline also because of the same reason (Figure 4).

One possible reason for the above mentioned changes to appear on CT images may be because of the long standing course of the condition. These symptoms may have developed in a course of 2 years and define inferring and destructive expansible nature of the condition as how they can cause disfigurement of the surrounding structures due to pressure without actually invading them.

According to our knowledge no such findings have been reported in literature where because of ameloblastoma of the mandible there is deflection of other surrounding structures specially the maxillary and those on the base of skull.

## 4. Conclusion

The basic objective of reporting such a case was to discuss the advantage of CT imaging in such cases to see the extent of lesion and the extent of destructive effect caused on the surrounding structures because of unusual expansion of lesion which clinically appears to be a routine case and also add to the review of literature for such unusual findings.
